# Diagnosis and management of osteoporosis in Saudi Arabia: 2023 key updates from the Saudi Osteoporosis Society

**DOI:** 10.1007/s11657-023-01242-w

**Published:** 2023-05-22

**Authors:** Yousef Al-Saleh, Riad Sulimani, Shaun Sabico, Fahad M. Alshahrani, Mona A. Fouda, Mohammed Almohaya, Salwa B. Alaidarous, Hazem M. Alkhawashki, Mohammed Alshaker, Hanan Alrayes, Najla Saleh, Nasser M. Al-Daghri

**Affiliations:** 1 Department of Endocrinology, Dr. Mohammad Alfagih Hospital, Riyadh, Saudi Arabia; 2 Department of Medicine, College of Medicine, King Saud University, King Saud University Medical City, Riyadh, Saudi Arabia; 3 Chair for Biomarkers of Chronic Diseases, College of Science, King Saud University, Riyadh, Saudi Arabia; 4 King Abdullah International Medical Research Center, Riyadh, Saudi Arabia; 5 College of Medicine, King Saud Bin Abdulaziz University for Health Sciences, Riyadh, Saudi Arabia; 6 Obesity, Endocrine and Metabolism Center, King Fahad Medical City, Riyadh, Saudi Arabia; 7 College of Medicine, King Saud Bin Abdulaziz University for Health Sciences, Jeddah, Saudi Arabia; 8 King Abdullah International Medical Research Center, Jeddah, Saudi Arabia; 9 Department of Medicine, King Abdulaziz Medical City, Jeddah, Ministry of National Guard Health Affairs, Jeddah, Saudi Arabia; 10 Advanced Medical Center, Riyadh, Saudi Arabia; 11 Department of Family Medicine and Polyclinic, King Faisal Specialist Hospital and Research Center, Riyadh, Saudi Arabia; 12 Prince Sultan Military Medical City, Riyadh, Saudi Arabia

**Keywords:** Diagnosis, Osteoporosis, Saudi Osteoporosis Society

## Abstract

***Summary*:**

The Saudi Osteoporosis Society (SOS) has updated its guidelines for the diagnosis and management of osteoporosis in Saudi Arabia (SA), with emphasis on postmenopausal women. This document is relevant to all healthcare professionals in SA involved in the care of patients with osteoporosis and osteoporosis-related fractures.

**Introduction:**

The SOS launched the first national osteoporosis guidelines in 2015 and spearheaded the Gulf Cooperation Council Countries (GCC) osteoporosis consensus report in 2020 which was under the auspices of the European Society for Clinical and Economic Aspects of Osteoporosis (ESCEO). This paper highlights a major update of the guidelines in the SA setting.

**Methods:**

This guideline is an adaptation of the current guidelines derived from ESCEO, the American Association of Clinical Endocrinologists (AACE), and the GCC osteoporosis consensus report and studies on osteoporosis done in SA. Where accessible, the timeliest systematic review, meta-analysis, and randomized controlled trials were used as evidence.

**Results:**

The present update includes new recommendations for the assessment of osteoporosis taking into consideration the Saudi model of FRAX for fracture probabilities, appropriate doses for the maintenance of vitamin D status and calcium, the use of representative blood analytes for therapy monitoring, the use of romosozumab and sequential therapy in the pharmacological management strategies, and the establishment of fracture liaison services to prevent secondary fractures.

**Conclusion:**

This updated guideline is for all healthcare professionals involved in osteoporosis and post-fracture care and management in SA and harmonized the most up-to-date changes in the field based on evidence-based medicine for use in the local setting.

**Supplementary information:**

The online version contains supplementary material available at 10.1007/s11657-023-01242-w.

## Introduction

Recommendations for the diagnosis and management of osteoporosis in Saudi Arabia (SA) can be traced back in 2004 with an updated version in 2011 [1, 2]. These recommendations were mainly perspectives from a group of local experts and implemented mostly from their affiliated institutions [1, 2]. The first official osteoporosis guideline was published by the Saudi Osteoporosis Society (SOS) in 2015 and was drafted by 14 key opinion leaders in the field [3]. At a regional level, the SOS, together with its counterparts, spearheaded the formation of osteoporosis guidelines/consensus report within the wider Pan-Arab region as well as the Gulf Cooperation Council (GCC) bloc [4, 5]. A key missing gap in all the documents mentioned is the lack of country-specific fracture risk assessment (FRAX) models. The scope of the present guidelines therefore is to update the previous local recommendations, taking into consideration the latest strategies in osteoporosis management, therapeutics available, and strategies for the prevention of fragility fractures in postmenopausal women and men above 50 years, within the SA setting. This article is only a reflection of the current state of osteoporosis within SA, and periodic revisions are essential due to the rapid developments in the field of bone health. The updated guideline is intended for all healthcare professionals in SA involved in the prevention or the management of osteoporosis and its complications. Local practitioners are encouraged to use the available information presented, taking into consideration the patient’s clinical information, individual circumstances and availability of local resources. The present guideline, prepared by the key members of SOS, was endorsed by the Saudi Health Council, Committee of National Societies of the International Osteoporosis Foundation (IOF) as well as the Saudi Rheumatology Society and the Saudi Society of Endocrinology and Metabolism.

## Background

Osteoporosis is a systemic skeletal disorder that significantly increases the individual’s risk of fracture, consequently affecting morbidity and mortality. While historically it can be argued that osteoporosis is as old as human civilization itself, it was only in 1994 when the World Health Organization (WHO) officially recognized it as a disease [6]. The acknowledgement from WHO gave osteoporosis the needed spotlight to be considered as another serious threat to global health, which, together with other age-related chronic diseases, go hand in hand with the substantially increasing elderly population. In parallel, a multi-national osteoporosis movement gave rise to the International Osteoporosis Foundation (IOF) in 1998, the largest non-governmental organization of its kind dedicated to promote bone and musculoskeletal health as a worldwide priority [7]. Prior to the founding of IOF, the first osteoporosis guideline was published in 1997 which was tailored to European settings [8], followed by its American counterparts, the National Institute of Health (NIH) panel of experts in 2001 [9], and the American Association of Clinical Endocrinologists (AACE) in 2010 [10]. Nation-specific guidelines started to emerge following the launch of a computer-based algorithm (FRAX) in 2008, which calculates the 10-year probabilities of a major osteoporotic fracture and hip fracture based from, initially, 8 country-specific models [11]. As of 2021, FRAX is available in 78 countries including SA, covering > 80% of the world population and included in >100 guidelines worldwide [12].

What makes osteoporosis of great clinical concern are the fractures that can arise from those most vulnerable, the elderly population. A recent meta-analysis of 86 studies (*N*=103,334,579 individuals aged 15–105) estimated that the over-all global prevalence of osteoporosis was 18.3% [13]. A separate meta-analysis of 40 studies (*N*=79,127) indicated that the prevalence of osteoporosis among older adults was 21.7%, with osteoporosis prevalence in the elderly women being almost three times higher than elderly men (35.3% versus 12.5%) [14]. Consequently, data from the most comprehensive analysis of the burden of fractures indicated that a total of 178 million new fractures was documented in 2019, with those aged 95 years and above having the highest age-specific rate of fracture at more than 15000 per 100,000 population [15]. Furthermore, for all age groups older than 64 years, females had substantially more fracture cases (as much as 54% higher among those 95 years and older) than males [15]. Cost of fragility fracture in Europe alone is expected to increase from €37.5 billion in 2017 to €47.4 billion in 2030 (an increase of 27% since 2017) [16].

In SA, the most recent systematic review on osteoporosis-related outcome indicated that a total of 174,225 osteoporosis-related fractures occurred in 2019 alone, translating to an estimated economic burden amounting to more than 2.3 billion Saudi riyals [17]. On the other hand, the number of hip fractures prospectively gathered in 15 local hospitals over a 2-year period among Saudis aged ≥ 50 was 2949 in 2017 and 2018 and is expected to increase by as much as 20,328 in 2050 [12]. This sevenfold increase in 2050 is anticipated if no drastic changes are done in the current osteoporosis management practice in SA. These figures equate to an annual incidence of 77.5/100,000 in women and 56.8/100,000 in men [12]. The probability of hip fracture in the Saudi population at the age of 50 is 4.6% in women and 3.7% in men, approximately half of the estimates from neighboring Kuwait and Abu Dhabi [12]. Given the anticipated burden of diseases, the Ministry of Health (MOH) in cooperation with SOS launched the National Plan for Osteoporosis Prevention and Management in April 2018 [18]. This national initiative has an overarching goal of making osteoporosis a national health priority focusing on five key areas: (a) education and health promotion; (b) screening, diagnosis, and treatment; (c) post-fracture care and secondary prevention; (d) self-management and falls prevention; and (e) research and evaluation [18].

## Saudi Osteoporosis Society Task Force

The Saudi Osteoporosis Society (SOS) hosted member experts and respective leaders in osteoporosis (YA, RA, FMA, MAF, MA, SBA, HMA, MA, HA ,and NS) from the major regions of SA, together with leaders from the Chair for Biomarkers of Chronic Diseases (CBCD) in King Saud University, Riyadh, SA (SS, NMA), to update the national guidelines for osteoporosis management. The guidelines update was first conceived on April 10, 2022, following the release of the Saudi version of FRAX (https://www.sheffield.ac.uk/FRAX/tool.aspx?country=84). The virtual assembly of experts commenced last June 8, 2022. Members of the task force were assigned topics pertinent to the osteoporosis update, with inspirations taken mostly from the GCC adaptation [5], the American Association of Clinical Endocrinologists (AACE) [19], and the most recent guidelines from the European Society for Clinical and Economic Aspects of Osteoporosis (ESCEO) [20]. Timelines and draft formulations were also discussed, taking into full consideration other relevant and timeliest guidelines, meta-analysis, systematic reviews and clinical trials from the national, regional and international levels. The first draft was circulated last September 5, 2022, and several revisions took place through correspondence until the final version for submission was accepted by all members last January 10, 2023.

## Diagnosis of osteoporosis

The operational cut-off used to diagnose osteoporosis is a BMD T-score of ≤ −2.5 in the femoral neck, lumbar spine, or distal radius [6, 19]. Following the 2020 guidelines of AACE, osteoporosis is also considered for those with low-trauma spine or hip fracture (regardless of BMD), a T-score of −1.0 and −2.5 with fragility fracture, or high FRAX based on Saudi-specific thresholds [19]. Osteoporosis screening is recommended for all Saudi women and men aged 60 and above, women ≥ 40 years with sustained low-trauma fragility fracture, or younger postmenopausal women with history of fragility fracture; premature menopause (<45 years), prolonged secondary amenorrhea, those on long-term glucocorticoid therapy, adults with primary hyperparathyroidism, and those with radiological evidence suggestive of fragility fracture, kyphosis or loss of height [3].

## Risk assessment

**Fig. 1:**
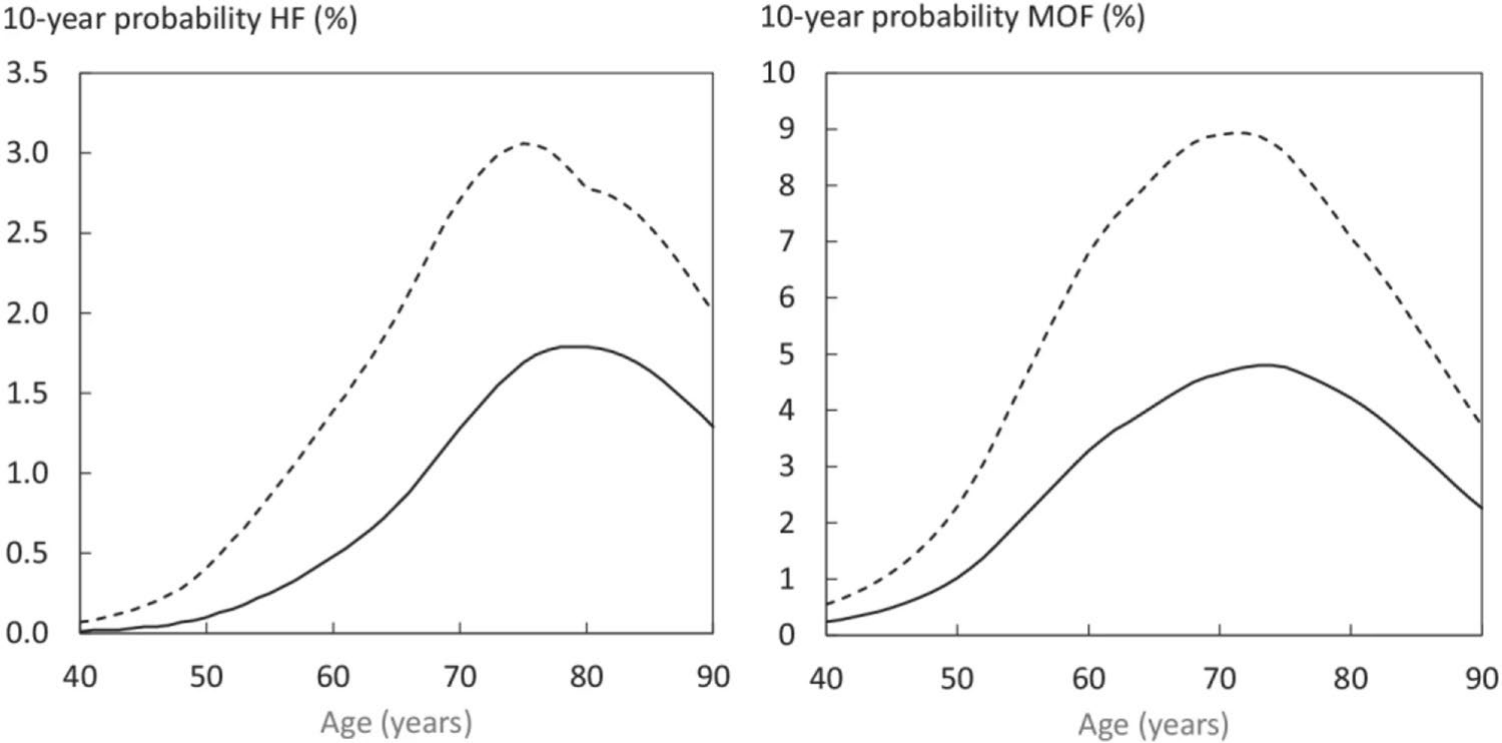
Ten-year probability of a major osteoporotic fracture in Saudi women by age [12]. The solid line shows probabilities in Saudi women with no clinical risk factors; dotted line shows probabilities in women with a prior fragility fracture. BMI was set at 25 kg/m^2^ (modified with permission from Saleh et al. (2022) (12))

A cluster of risk factors have been identified, established, and condensed into a single diagnostic tool known as FRAX®, developed in the World Health Organization (WHO) Collaborating Centre for Metabolic Bone Diseases at the University of Sheffield, UK, to predict fracture risk independent of BMD [11]. These clinical risk factors include country (of birth), age, sex, body mass index, smoking, alcohol intake, glucocorticoid treatment, rheumatoid arthritis, previous fracture, parental history of fracture, BMD, and diseases strongly associated with secondary osteoporosis (Supplementary Table 1) [11]. Fracture risk assessment in SA has used the FRAX US version (white Caucasian) until 2021. As of 2022, country-specific FRAX calculator for SA is now available online; https://www.sheffield.ac.uk/FRAX/tool.aspx?country=84. Ten-year probabilities of hip and major fracture for Saudis which was set at BMI 25kg/m^2^ are shown in Fig. 1 [12]. The FRAX model was based from collected data on low-energy hip fracture during 2017–2018 from 15 hospitals across SA and limited to Saudi citizens. A limitation of the Saudi FRAX model is that it was based on approximately only 12% of the at-risk population, which means the current model may not be generalizable. The obtained hip fracture rates however were similar to those obtained from other GCC counterparts, UAE and Kuwait, but not Qatar [21].

Probability-based assessment of fracture risk using age-specific intervention thresholds has also been developed for Saudi women with BMI 30kg/m^2^ as shown in Table 1 [22]. For instance, for a Saudi woman aged 70 years, BMD is not recommended if the fracture probability is below 3.96% (low risk). BMD is recommended for a Saudi woman having the same age if the probability falls between 3.96 and 9.1%. Treatment is recommended for a Saudi woman of the same age without the need for BMD testing if the probability exceeds 9.1%.

**Table 1:** Ten-year probability of a major osteoporotic fracture (MOF) and hip fracture (HF) by age at the intervention threshold and lower and upper assessment thresholds calculated with FRAX for SA [22]

Age (years)	Intervention thresholds ^a^ (%)		Assessment threshold			
			Lower ^b^ (%)		Upper ^c^ (%)	
	MOF	HF	MOF	HF	MOF	HF
40	0.48	0.05	0.21	0.01	0.58	0.06
45	0.97	0.13	0.43	0.03	1.16	0.16
50	1.97	0.32	0.89	0.08	2.36	0.38
55	3.88	0.66	1.81	0.19	4.66	0.79
60	5.86	1.08	2.84	0.37	7.03	1.30
65	7.00	1.54	3.51	0.62	8.40	1.85
70	7.58	2.10	3.96	0.99	9.10	2.52
75	7.25	2.36	4.01	1.30	8.70	2.83
80	5.90	2.13	3.49	1.37	7.08	2.56
85	4.50	1.93	2.68	1.24	5.40	2.32
90	3.00	1.51	1.80	0.97	3.60	1.81

Thresholds presented in percentages (%). ^a^The threshold is the probability of a MOF for a woman with BMI 30 kg/m^2^ and a previous fracture and no other clinical risk factors without BMD. ^b^The lower assessment is the probability of a MOF for a woman with BMI 30 kg/m^2^ and no clinical risk factors without BMD. ^c^The upper assessment was set at 1.2 times the intervention threshold [22]

**Fig. 2:**
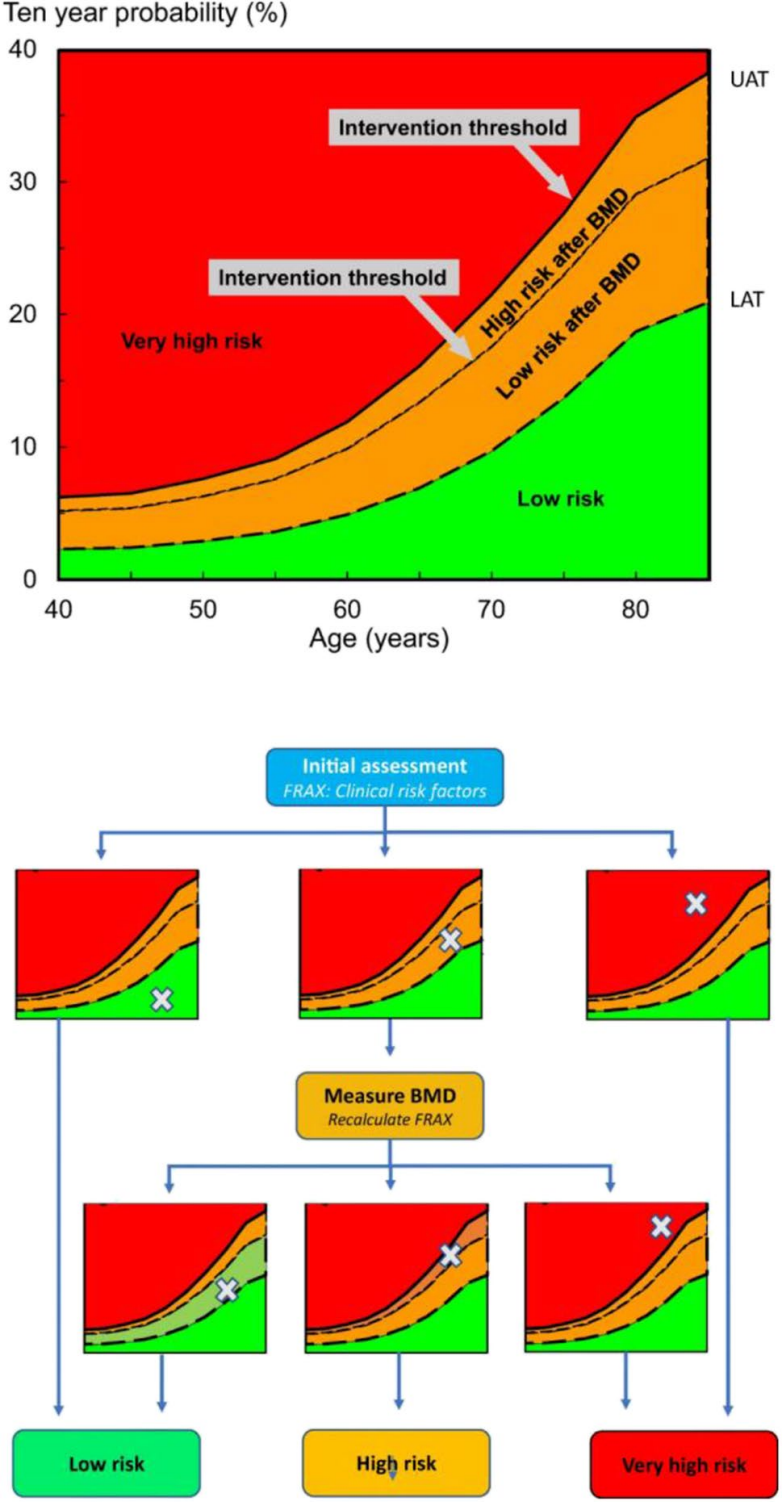
Categorization of fracture risk using FRAX major osteoporotic fracture probability in postmenopausal women as recommended by IOF and ESCEO [23]. Initial risk assessment uses FRAX with clinical risk factors without BMD. If FRAX probability falls in the intermediate (orange) zone, then BMD assessment is needed with recalculation of FRAX probability after including femoral neck BMD. Treatment is based on recalculated risk

Figure 2 outlines the algorithm for characterization of fracture risk by FRAX with risk factors alone without the need to do BMD initially as recommended by IOF and ESCEO [40]. This approach helps also to minimize the number of patients who do BMD. From the initially obtained Saudi FRAX scores in the absence of BMD, physicians may be able to categorize risk as follows:

- Patients who fall in the green zone, below the lower assessment threshold (LAT) line, are at low risk and do not require BMD measurement. They need advice about general bone health.
- Patients who fall in the red zone, above the upper assessment threshold (UAT) line, should be treated. BMD is done as baseline to monitor treatment progress.
- Patients who fall in the orange zone between the LAT and UAT lines are at intermediate risk and must undergo BMD measurement.

These later patients FRAX score should be recalculated after doing BMD. Those whose score fall above the intervention threshold line should be treated. While those who fall below that line is at low risk do not need treatment. The above risk stratification applies to both men and women.

## Bone turnover markers for osteoporosis

The use of bone turnover markers in clinical practice was not mentioned in the first Saudi osteoporosis guidelines, given its limited clinical application. Over time, major international organizations such as the IOF and the International Federation of Clinical Chemistry and Laboratory Medicine (IFCC) recommended 1 marker for bone formation, serum Procollagen type 1 N Propeptide (s-PINP), and one marker for bone resorption, serum C-terminal cross-linking telopeptide of type 1 collagen (s-CTX), as reference analytes to predict fracture risk [24]. While accumulated evidence reveals that these markers of bone turnover still cannot be used to diagnose osteoporosis and assess fracture risk until standardization is finalized, its clinical utility is suggested for monitoring treatment response and adherence for those on bisphosphonate therapy, as well as adjunct markers to improve detection of secondary osteoporosis [24]. Other blood biomarkers, including 25(OH)D, intact PTH, and calcium and hormone profile, among others, are used for detecting secondary causes of osteoporosis, which are largely dependent on the disorders being ruled out by the attending healthcare specialist (Supplementary Table 2). Proper sampling precautions such as morning timing of samples and the fasting state of the patient should be exercised in using BTMs to avoid misleading results.

## Non-pharmacological management

Calcium supplementation is recommended if dietary calcium intake is below 800mg. Daily calcium intake should be between 800 and 1200mg which can be achieved through dietary sources, based on the European guidance from the IOF and ESCEO [20]. Calcium intake for the present guideline is almost 50% lower than the original guideline, which recommended 1500mg/day for those above 50 years, men or women [3].

Vitamin D supplementation in the form of cholecalciferol is also recommended for all individuals with vitamin D deficiency [25(OH)D <50nmol/L)], with maintenance doses starting from 1000 to 2000IU/day once the target (>50nmol/l) is achieved for the general population, according to the recent guidelines from the GCC Vitamin D Advisory group and the SOS. The current vitamin D dose is twice as high as the original guideline, which recommended only 600–1000IU/day for postmenopausal women and men above 50 years [3]. Certain subjects like those with obesity, malabsorption syndrome, or dark skin may need higher maintenance doses of up to 4000 IU Daily. At present, although vitamin D deficiency remains very common in the general population of SA, the prevalence has begun to drop significantly from 2008 to 2017, particularly among adults above 40 years (86.2% to 64.7%), with an over-all circulating 25(OH)D increase of 2.2 nmol/year during the same period [25]. Achieving and maintaining 25(OH)D levels of 75nmol/l (30 ng/ml) is recommended for frail, osteoporotic, and older patients based on the experts’ consensus for the management of vitamin D in SA [26]. For postmenopausal women who are vitamin D sufficient, sun exposure for 15 min/day 3–4 times weekly is recommended [26, 27].

Aside from the supplements mentioned above, adequate protein intake (US recommended daily allowance of 1 g/kg) is recommended to decrease bone loss especially among those with previous hip fracture [19]. Finally, regular weight-bearing activities and exercise are encouraged for strength and balance to prevent falls and decrease fracture risk [20]. Physical activities should be tailored to the patient’s individual capacity and needs.

## General pharmacological management

**Fig. 3:**
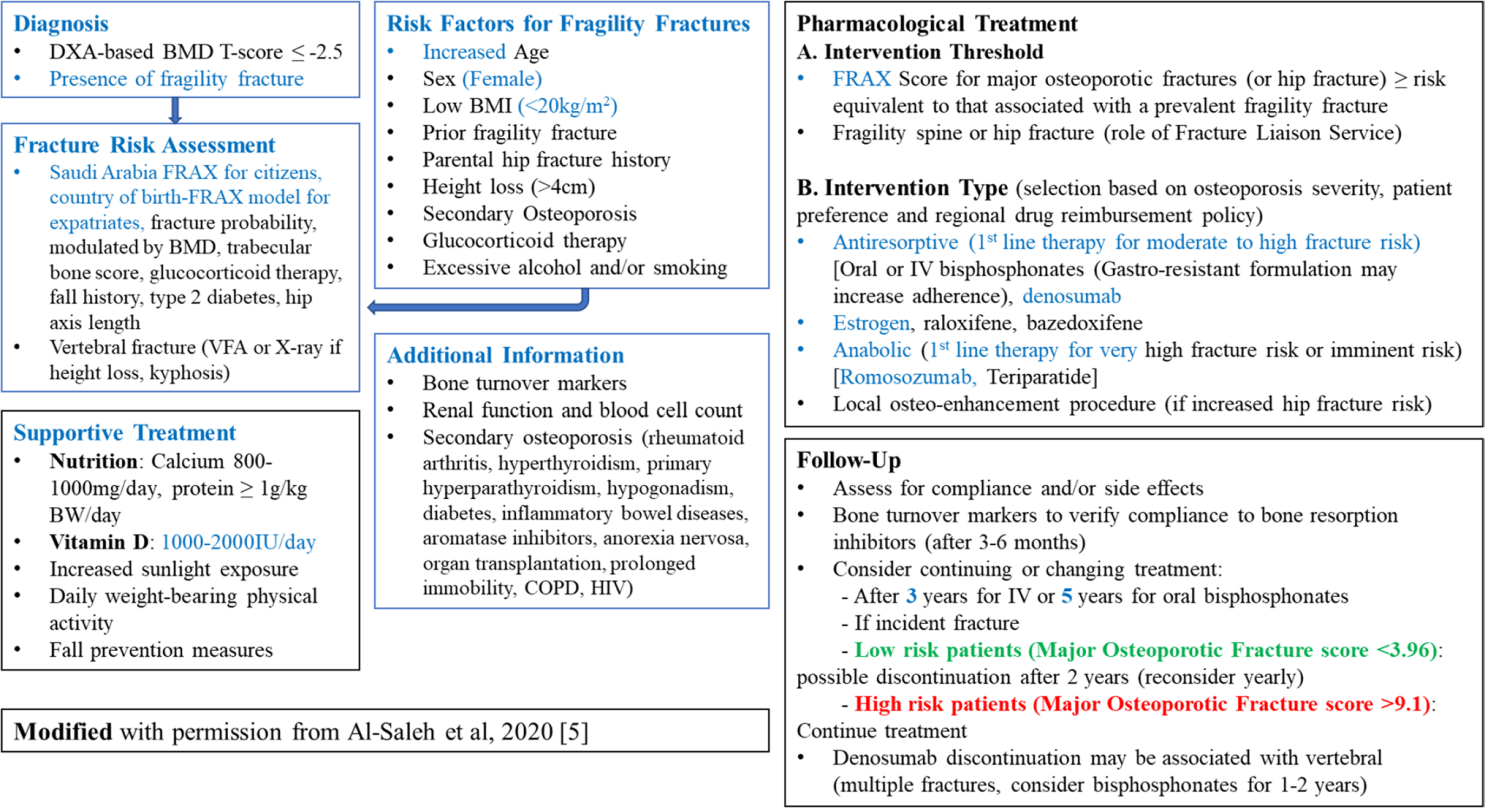
Diagnosis and management of osteoporosis

A summary of pharmacological treatment options is provided in Table 2 which is a modified version of the recent regional recommendations from the GCC bloc [5], based on the guidance of IOF and ESCEO [20]. Romosozumab has been added as a new anabolic drug for the first-line treatment for those with very high risk of fracture. A schematic diagram on the updated over-all diagnosis and management of osteoporosis is presented in Fig. 3.

**Table 2:** Pharmacological management of osteoporosis

Category	First-line therapy	Alternative first-line therapies
Young postmenopausal with only vertebral osteoporosis and history of or at high risk for breast cancer	Raloxifene Falling out of favor due to the presence of more effective and safer medications	Oral bisphosphonates IV bisphosphonates Denosumab Teriparatide
Older postmenopausal/or younger but with concerns of hip fracture	Oral bisphosphonates* Denosumab	IV bisphosphonate Teriparatide Romosozumab
Osteoporosis in men	Oral bisphosphonates* Denosumab	IV bisphosphonate Teriparatide
Severe osteoporosis Very low BMD T≤3.0 + one fracture or ≤2.5 + 2 fractures OR Imminent fracture risk (in the immediate post-fracture period)	Teriparatide Romosozumab	Oral bisphosphonates IV bisphosphonate Denosumab
Glucocorticoid-induced osteoporosis (GIOP)	Oral bisphosphonates IV bisphosphonate Denosumab Teriparatide	

*Oral bisphosphonate (only alendronate or risedronate has evidence (Ia) for hip fracture efficacy), IV bisphosphonate (zoledronic acid). Alternative first line is considered if first line were not feasible, contraindicated, or failed. Can also be considered when therapy has to be discontinued but patient still requires treatment

## Bisphosphonates

Bisphosphonates (alendronate, ibandronate, risedronate, and zoledronate) are powerful inhibitors of bone resorption via decreased activity of osteoclasts and is considered as first-line treatment for the majority of postmenopausal women with osteoporosis [28]. Its clinical application in patients with acute fracture however remains controversial. A recent meta-analysis involving 16 studies (*N*=5022] showed that while bisphosphonates had no impact in fracture healing time, it significantly influences favorable increase in BMD, which suggests that appropriate individuals may benefit from early bisphosphonate treatment following injury [29]. Major guidelines acknowledge the favorable safety profile of bisphosphonates, but also recommend periodic assessment of fracture risk, depending on the route of treatment [19, 23]. Bisphosphonates are contraindicated in patients with low GFR, below 35 ml/min.

## Parathyroid hormone (PTH) analogs

Anabolic agents such abaloparatide (modified PTH-related peptide 1–34) and teriparatide (recombinant human PTH1–34) increase bone formation and are recommended for osteoporotic individuals with high fracture risk or unresponsive to other therapies [19]. Teriparatide, in particular, is effective in reducing the risk of both vertebral and non-vertebral fractures but not hip fracture [23]. These classes of drugs are not advised for individuals with higher-than-normal increase in bone turnover, such as those observed in metabolic bone diseases (except primary osteoporosis). Data from the VERtebral fracture treatment comparisons in osteoporotic women (VERO) trial demonstrated the efficacy of teriparatide over risedronate in reducing the incidence of imminent fractures in postmenopausal women with recent fractures after a 24-month treatment (56% for vertebral fractures and 52% for clinical fractures compared to risedronate) [30]. On the other hand, abaloparatide also effectively reduces relative risk of vertebral and non-vertebral fracture as well as risk of fractures in postmenopausal women with osteoporosis compared to other treatment options in a recent network meta-analysis [31]. PTH analog drugs are recommended as 2-year treatment course. [32].

## Selective estrogen receptor modulators (SERMs)

SERMs are primarily indicated for the prevention of breast cancer in postmenopausal women with osteoporosis, among which raloxifene is the most commonly prescribed and widely available [19, 23]. In a recent network Bayesian meta-analysis involving 9 randomized trials (*N*=60,732), raloxifene, as compared to other SERMs, was more potent in terms of decreasing risk of invasive breast cancer by as much as 35% [odds ratio (OR) 0.65, 95% confidence interval (CI) 0.48–0.85), while tamoxifen increased the risk for endometrial cancer (OR 2.42, 95% CI 1.10–7.35) [33]. Raloxifene is also beneficial, relative to placebo, in terms of decreasing vertebral fracture risk, not hip fracture, with reported cardiovascular benefits but with increased risk for thromboembolic events [34, 35]. The use of raloxifene, however, is quite low in SA since there are more effective and safer alternatives.

## Molecular-targeted drugs: denosumab and romosozumab

Denosumab is a fully human monoclonal antibody that inhibits the cytokine RANKL (receptor activator of nuclear factor kappa B ligand), essentially blocking osteoclast maturation and consequently reducing bone resorption [36]. Denosumab has an established profile in fracture risk reduction and more recently in significant reduction of falls, based on a pooled analysis of 10,036 individuals, 5030 of whom received denosumab (hazard ratio = 0.79; 95% CI 0.66–0.93; *p* = 0.0061) [37].

On the other hand, romosozumab is a humanized monoclonal antibody that enhances bone formation while reducing bone resorption through binding and inhibition of sclerostin [38]. Both the European guidelines and AACE recommend 1-year use of romosozumab for the treatment of patients with very high risk of fracture as sequential treatment [22, 39], with the latter considering romosozumab as a “rescue drug” for postmenopausal women with very high fracture risk [22]. Adverse and serious events with romosozumab were no different compared to placebo and other osteoporosis drugs in a recent meta-analysis involving 10 eligible studies (*N*=6137) in the romosozumab group versus *N*=5732 in the control group) [40]. Romosozumab is not approved for men with osteoporosis.

A complete list of osteoporosis drugs available for prevention and management as endorsed by the Saudi Food and Drug Administration, SFDA, is provided in Table 3.

**Table 3:** Approved drugs by the SFDA for postmenopausal osteoporosis management

Drug	Treatment dose	Duration
Alendronate (Fosamax)	70 mg PO weekly ^A^ 70 mg + vitamin D	5 years
Denosumab (Prolia)	60 mg SQ every 6 months	Unlimited
Raloxifene (Evista)	60 mg PO daily	10 years
Romosozumab (Evenity)	210 mg SQ monthly	One year
Teriparatide (Forteo) (Bonteo: biosimilar available in KSA)	20 µg SQ daily	2 years, course can be repeated
Zoledronate (Reclast, generic)	5 mg IV once yearly	3 years

*IV*, intravenous; *PO*, oral; *SQ*, subcutaneous. ^A^Available as tablet and unit dose liquid; modified with permission [19]

**Fig. 4:**
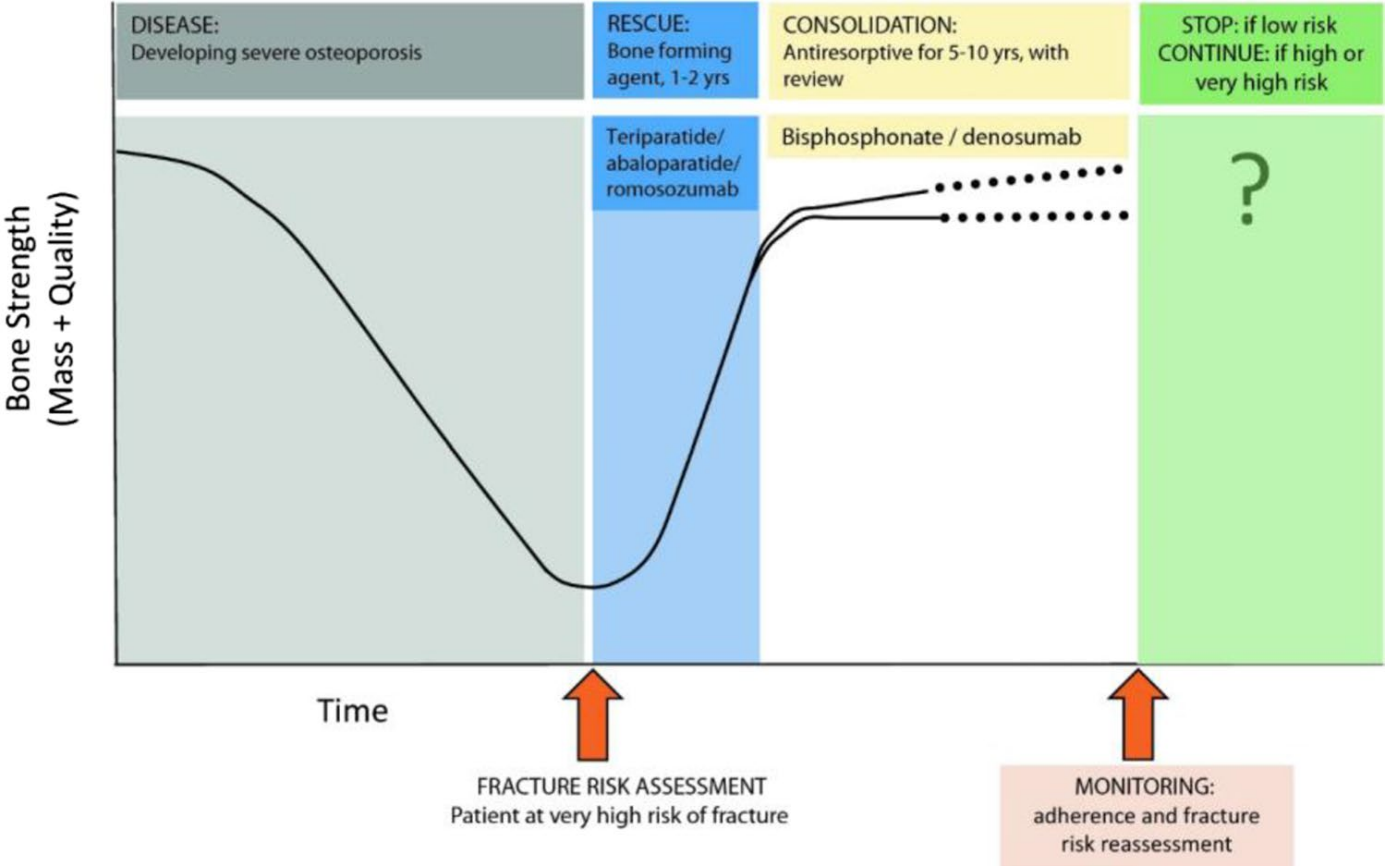
Proposed sequential therapy for patients with severe osteoporosis or imminent risk of fracture, reproduced with permission [39]

For patients with very high fracture risk, a pragmatic approach has been recommended by the ESCEO, which entails initial therapy with a bone-forming agent, followed by a consolidation period of antiresorptive therapy (sequential therapy) [22, 39]. Figure 4 demonstrates the outline of sequence of therapy for patients with severe osteoporosis with very high risk of fracture (markedly reduced BMD readings and multiple fractures, patients in the immediate post-fracture period, markedly increased FRAX score).

## Definition of successful therapy for osteoporosis

Management of osteoporosis, pharmacologic and non-pharmacologic, is geared towards successful treatment, of which the SOS adapts the description used by the AACE, which states that successful treatment meant stable or increasing bone mineral density, with no evidence of new fractures or vertebral fracture progression as a response to therapy for osteoporosis [19].

## Fracture liaison services

Fracture liaison services (FLSs) are generally promoted as the most suitable strategy that covers all aspects of secondary fracture prevention, including identification, education, risk evaluation, monitoring, and treatment [41]. Data from the most recent systematic review of 16 eligible studies from 2010 to 2020 indicated that FLS care was associated with a significantly lower probability of subsequent fractures (OR: 0.70, 95% CI: 0.52–0.93, *p*=0.01) [41] and significant reduction in cost. In SA, retrospective data gathered from a local major tertiary hospital in 2008–2012 showed that very few patients receive osteoporosis-specific therapy among those surgically treated, with more than 20% death rate 1 year after hip surgery [42]. Similar findings were found in a study done in another tertiary-center where post-fracture care was not optimal especially among men [43]. Worthy to note is that the first Saudi guidelines in 2015 did not have recommendation for FLS, but over time, this has been included in regional consensus statements [4, 5]. At present, only few institutions in SA provide FLS. It is considered a critical missing gap in the prevention of recurrent fractures and is currently a priority of the National Plan for Osteoporosis Prevention and Management in Saudi to establish more FLS and rehabilitation centers [18].

## Conclusion

The present document highlights the updated recommendations of the SOS in the diagnosis and management of osteoporosis in SA. These includes the use of the now available country-based FRAX, higher dose of vitamin D and calcium for postmenopausal women, the introduction of romosozumab in the pharmacological treatment of postmenopausal osteoporosis, and the establishment of FLS for post-fracture care. SA practitioners in the field of osteoporosis are encouraged to follow the updated guidelines. Future revisions in the guidelines will take into consideration the meta-analysis findings of multiple international cohorts to update the FRAX prediction tool [44].

### Supplementary information

Below is the link to the electronic supplementary material.

Supplementary file1 (DOCX 15 KB)
