# Supplementary material for: Diagnosis and management of osteoporosis in Saudi Arabia: 2023 key updates from the Saudi Osteoporosis Society
Source: Arch Osteoporos. 2023 May 22;18(1):75. doi: 10.1007/s11657-023-01242-w (PMC10202978; doi:10.1007/s11657-023-01242-w)
Supplement: Supplementary file 1 — Supplementary file1 (DOCX 15 KB) [file 11657_2023_1242_MOESM1_ESM.docx]

**Supplementary table 1**. Common Causes of Secondary Osteoporosis

| Endocrinopathies | Hyperthyroidism, Cushing syndrome, hypogonadism, hypopituitarism, primary hyperparathyroidism, diabetes mellitus, eating disorders, growth hormone deficiency, acromegaly |
| --- | --- |
| Gastrointestinal disorders | Celiac disease, inflammatory bowel disease, gastric bypass surgery, hemochromatosis and chronic liver diseases |
| Hematological disorders | Monoclonal gammopathy of uncertain significance, multiple myeloma, systemic hemochromatosis, beta thalassemia major, HIV |
| Autoimmune disorders | Rheumatoid arthritis, systemic lupus erythematosus, ankylosing spondylitis, multiple sclerosis |
| Renal disease | Renal tubular acidosis, chronic kidney disease |
| Medications | Corticosteroids, thyroid hormone, aromatase inhibitors, medroxyprogesterone acetate, GnRH agonists and antagonists, selective serotonin reuptake inhibitors, carbamazepine, phenytoin, cyclosporine, tacrolimus, antiretroviral therapy, lithium, heparin, furosemide and proton pump inhibitors |

**Supplementary table 2**. Biochemical assessment of secondary osteoporosis (These are done based on specific patient condition/ assessment)

| 24-hour urine calcium  25-hydroxyvitamin D  Albumin  Alkaline phosphatase  Bone resorption and formation markers  Calcium, Phosphate, Magnesium  Complete Blood Count (CBC)  Dexamethasone suppression test or 24-hour urinary free cortisol  Estradiol (in women)  Erythrocyte Sedimentation Rate (ESR)  LH, FSH and prolactin  Parathyroid hormone, intact (PTH)  Serum and urine protein electrophoresis  Serum ferritin  Morning Testosterone (in men)  Thyroid-stimulating hormone (TSH)  Tissue transglutaminase antibodies |
| --- |
